# Supplementary material for: The effects of omega-3 fatty acids on diabetic nephropathy: A meta-analysis of randomized controlled trials
Source: PLoS One. 2020 Feb 11;15(2):e0228315. doi: 10.1371/journal.pone.0228315 (PMC7012392; doi:10.1371/journal.pone.0228315)
Supplement: S1 Appendix — (DOCX) [file pone.0228315.s002.docx]

**Supporting Information**

S1 Appendix

The following MEDLINE subject headings (MeSH) search terms were used to search for trials within MEDLINE (PUBMED):

("Fish Oils"[Mesh] OR alpha Linolenic[tiab] OR Docosahexae*[tiab] OR Docosahexeno*[tiab] OR Eicosapentaenoic[tiab] OR Eicosapentanoic[tiab] OR fish oil*[tiab] OR omega 3[tiab] OR DHA[tiab] OR Docosahexaenoic Acid*[tiab] OR Eicosapentaenoic Acid*[tiab] OR EPA[tiab] OR Linolenate[tiab] OR Linolenic Acid[tiab] OR n3 Fatty Acid*[tiab] OR n3 Polyunsaturated[tiab] OR n3 PUFA[tiab] OR n 3 Fatty Acid*[tiab] OR n 3 Polyunsaturated[tiab] OR n 3 PUFA[tiab] OR Timnodonic Acid*[tiab]) AND ("Diabetes Mellitus"[Mesh] OR diabetes[tiab] OR diabetic[tiab] OR diabetics[tiab] OR "Diabetic Nephropathies"[Mesh]) AND ("Albuminuria"[Mesh] OR "Glomerular Filtration Rate"[Mesh] OR "Proteinuria"[Mesh:noexp] OR "Renal Insufficiency, Chronic"[Mesh] OR albuminuria[tiab] OR chronic kidney[tiab] OR chronic renal[tiab] OR glomerular filtration[tiab] OR proteinuria[tiab]) AND ("clinical trials as topic"[mesh] OR "clinical trial"[pt] OR random[tw] OR randomized[tw] OR "random allocation"[mesh]).
