# Supplementary figures and images for: The effects of omega-3 fatty acids on diabetic nephropathy: A meta-analysis of randomized controlled trials
Source: PLoS One. 2020 Feb 11;15(2):e0228315. doi: 10.1371/journal.pone.0228315 (PMC7012392; doi:10.1371/journal.pone.0228315)

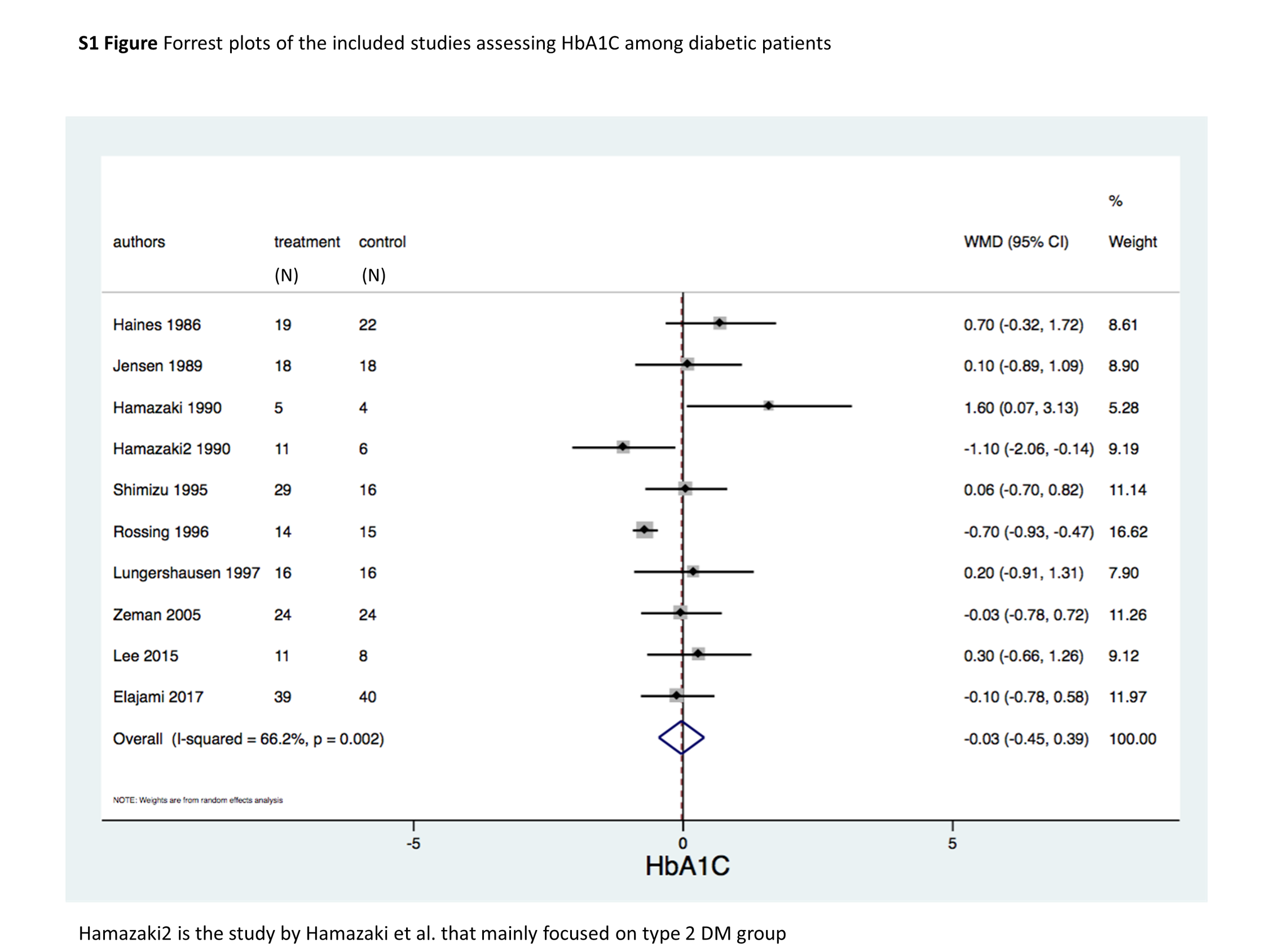

Supplement: S1 Fig — (TIF) [file pone.0228315.s003.tif]

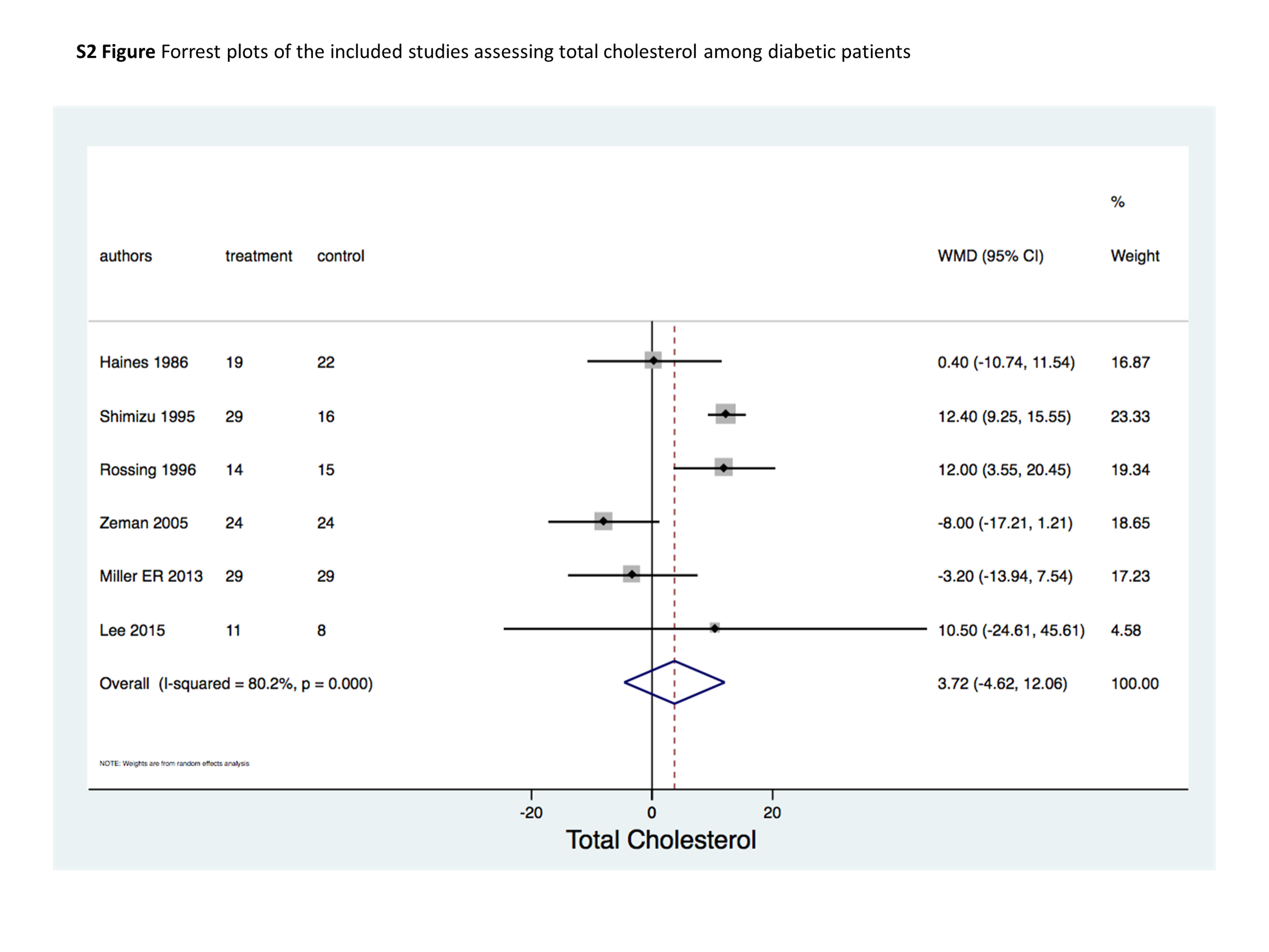

Supplement: S2 Fig — (TIF) [file pone.0228315.s004.tif]

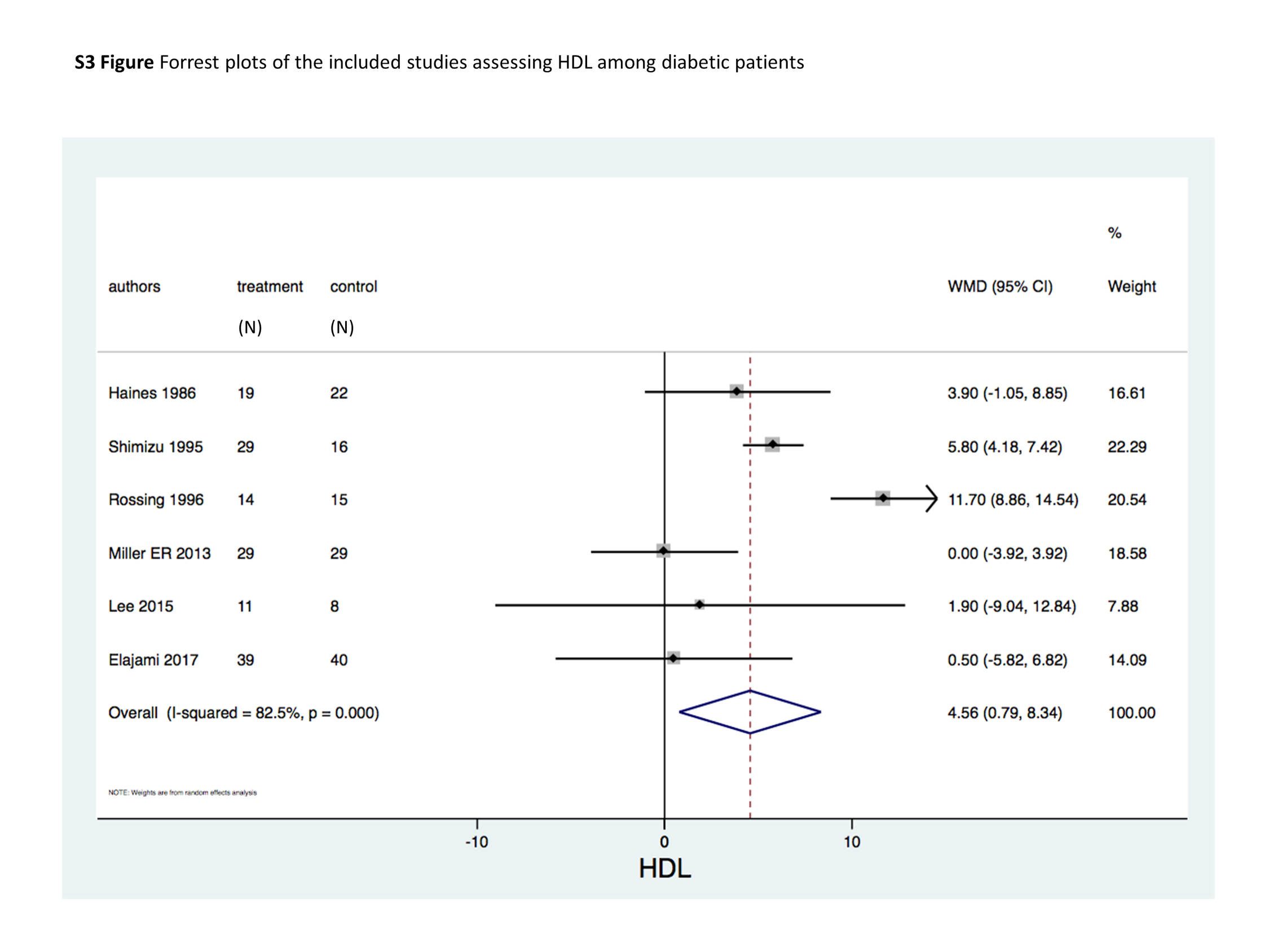

Supplement: S3 Fig — (TIF) [file pone.0228315.s005.tif]

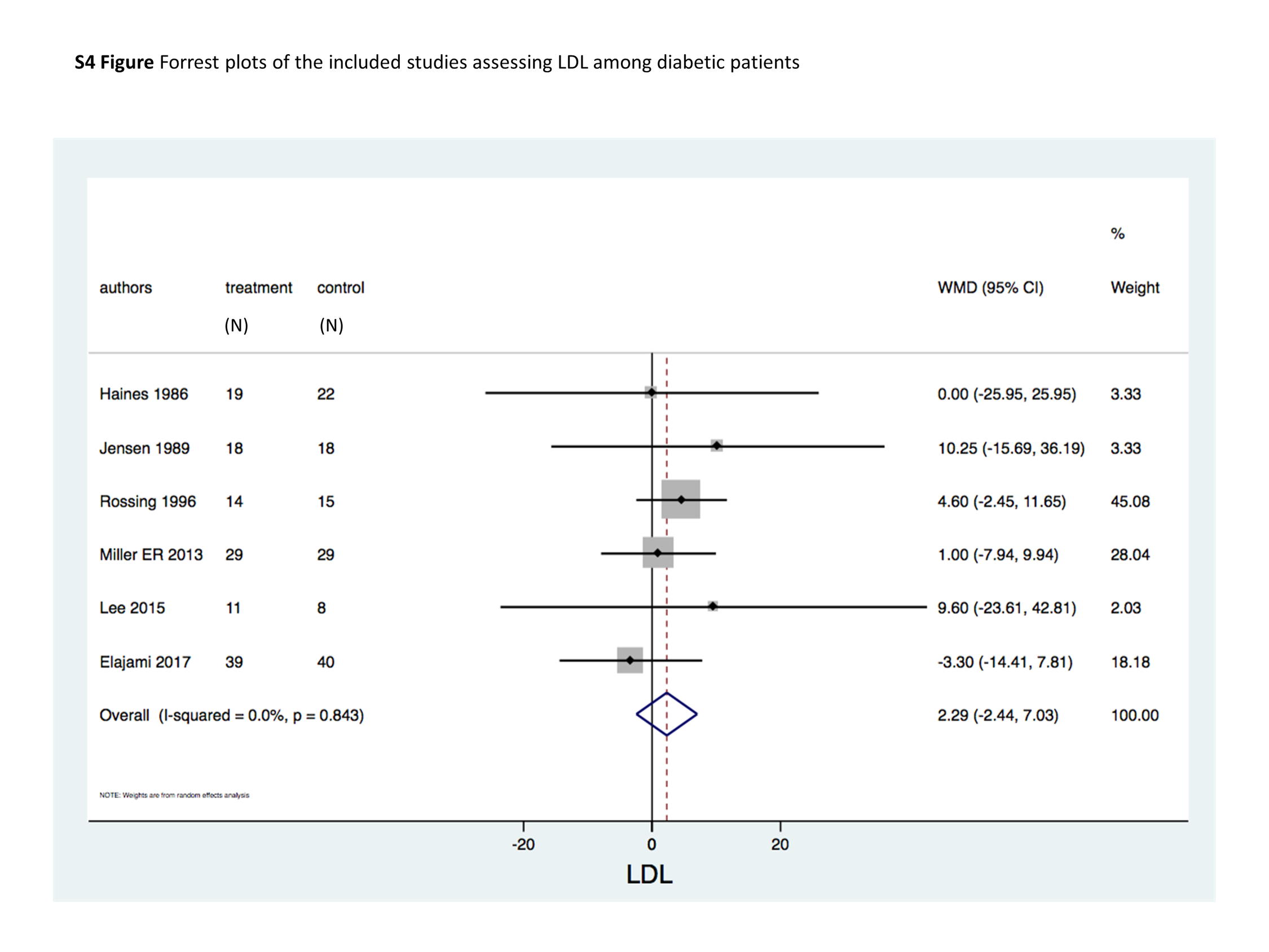

Supplement: S4 Fig — (TIF) [file pone.0228315.s006.tif]

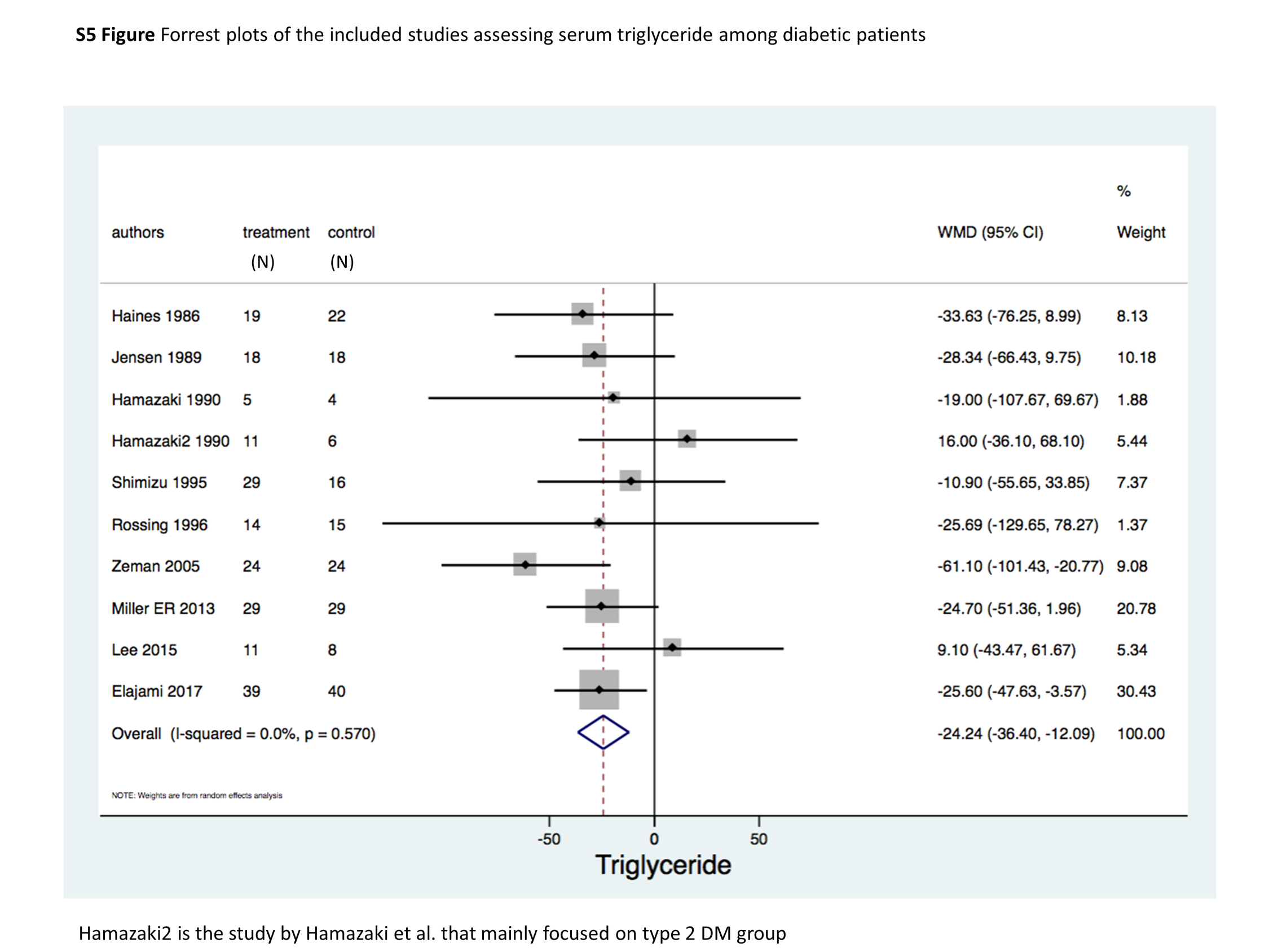

Supplement: S5 Fig — (TIF) [file pone.0228315.s007.tif]

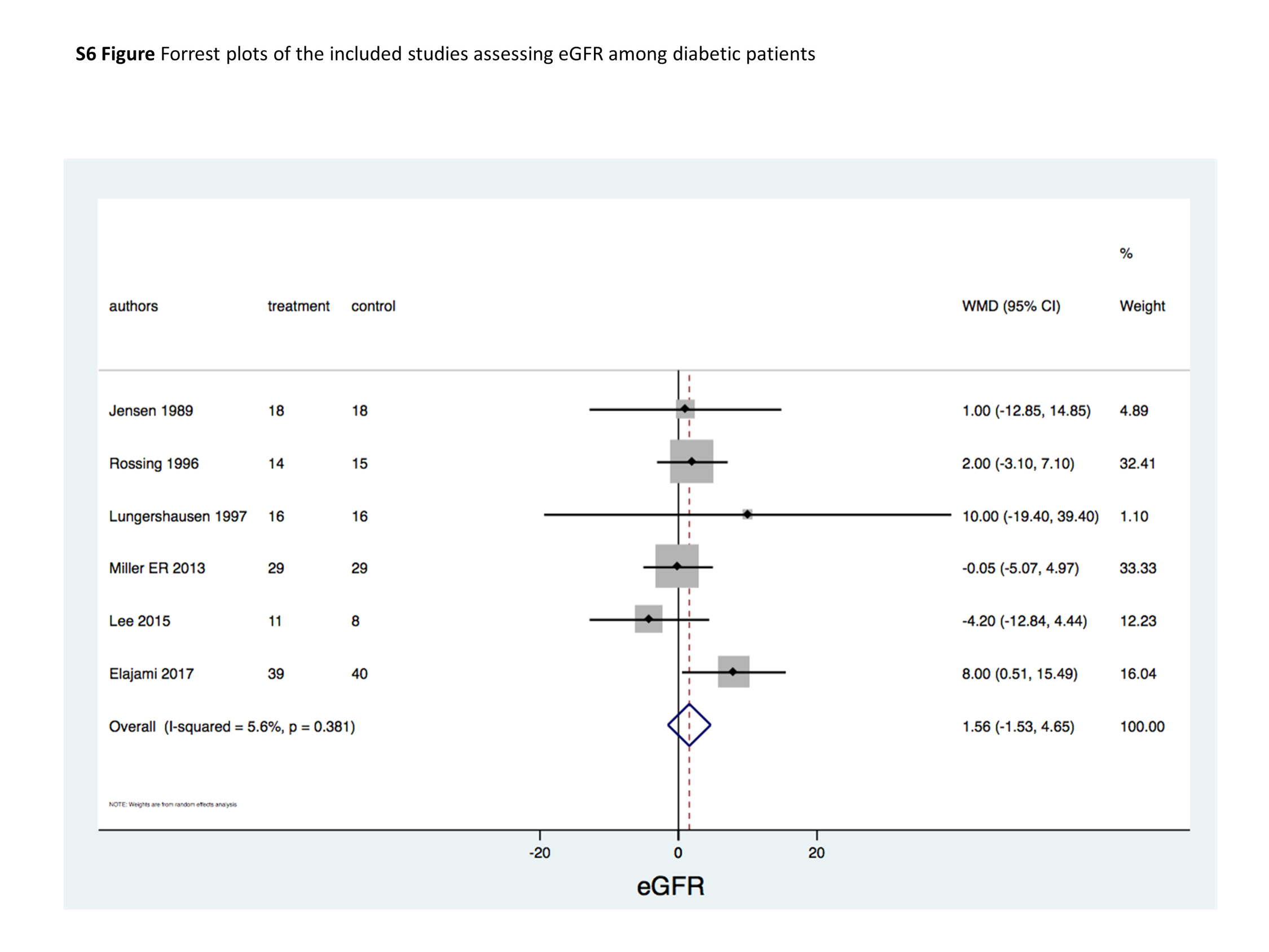

Supplement: S6 Fig — (TIF) [file pone.0228315.s008.tif]
