# Supplementary material for: The effects of omega-3 fatty acids on diabetic nephropathy: A meta-analysis of randomized controlled trials
Source: PLoS One. 2020 Feb 11;15(2):e0228315. doi: 10.1371/journal.pone.0228315 (PMC7012392; doi:10.1371/journal.pone.0228315)
Supplement: S1 Table — (DOCX) [file pone.0228315.s009.docx]

**Supporting Information**

S1 Table Summary effects of omega-3 fatty acids on outcomes of interest among diabetic patients (fixed effects model)

| Outcomes | No of study arms | No of patients | Weighted mean difference/Standardized mean difference* | Confidence interval | I^2^ | P-value |
| --- | --- | --- | --- | --- | --- | --- |
| Proteinuria | 11 | 342 | -0.19* | (-0.38, 0.01) | 0% | 0.06 |
| eGFR | 6 | 208 | 1.54 mL/min/1.73 m^2^ | (-1.40, 4.48) | 5.6% | 0.31 |
| SBP | 10 | 318 | -2.10 mmHg | (-4.48, 0.28) | 0% | 0.08 |
| DBP | 10 | 318 | 2.10 mmHg | (0.57, 3.63) | 39.8% | 0.007 |
| Triglyceride | 10 | 313 | -24.24 mg/dL | (-36.40, -12.10) | 0% | **<0.001** |
| TC | 6 | 168 | 9.07 mg/dl | (6.44, 11.71) | 80.2% | **<0.001** |
| HDL-c | 6 | 242 | 5.97 mg/dl | (4.73, 7.22) | 82.5% | **<0.001** |
| LDL-c | 6 | 215 | 2.29 mg/dL | (-2.45, 7.03) | 0% | 0.34 |
| HbA1C | 10 | 313 | -0.42% | (-0.60, -0.24) | 66.2% | **<0.001** |
