# Supplementary material for: The effects of omega-3 fatty acids on diabetic nephropathy: A meta-analysis of randomized controlled trials
Source: PLoS One. 2020 Feb 11;15(2):e0228315. doi: 10.1371/journal.pone.0228315 (PMC7012392; doi:10.1371/journal.pone.0228315)
Supplement: Attachment — Submitted filename: R2_Omega-3.docx [file pone.0228315.s011.docx]

January 6, 2020

**Re:	Manuscript entitled “The Effects of Omega-3 Fatty Acids** **on Diabetic Nephropathy: A Meta-analysis of Randomized Controlled Trials ”**

Submission ID: PONE-D-19-24289

Dear Editor,

Thank you for the thoughtful input and review of our manuscript. We believe as a result of this review, our study will have more value for your readers. We revised the manuscript based on the reviewers’ suggestions. We have attached our point by point response.

Thank you for your time and consideration. If you have any additional questions or comments, please let us know.

Sincerely,

Api Chewcharat, MD, MPH

Department of Epidemiology, Harvard T.H. Chan School of Public Health, Boston, MA 02115, USA

Email: Api.che@hotmail.com

**Responses to reviewer#2**

Reviewer #2: Dear Authors,

These details are required:
[1] Table 1 should provide eGFR data

Response: Thank you for your suggestion. We added eGFR data in table 1.

[2] Check reference citation...eg. No.4 is it WE Mitch?? should be.

Response: We appreciated the reviewer’s valuable input. We found the suggested reference very helpful to support our introduction. Hence, we added the reference as suggested.

**Responses to reviewer#3**

Reviewer #3: The authors have answered all the questions. However, they still should make a further discussion for the following question in the revised manuscript.
1. The reference 37 is not a meta-analysis, and did not support the related discussion. Please check the order of the references.

Response: Thank you for your comment. To the best of our knowledge, there were no other meta-analyses to support our findings as to why there was only significant reduction in proteinuria only among type 2 diabetes. We postulated that because in type 2 DM, there are higher inflammatory cytokines generated from adipose tissue compared to type 1 DM. Omega-3 fatty acids might help diminish these inflammatory cytokines that eventually leads to lower proteinuria in only type 2 DM. However, we did not have enough information to confirm this hypothesis since the inflammatory markers were not available in many trials that we included. Moreover, it might be explained by the inadequate power to detect the significant difference of proteinuria among type 1 DM because there were only 97 participants in type 1 DM compared to 213 participants for type 2 DM. Future studies are needed to assess whether omega-3 fatty acids could reduce the inflammatory cytokines differently between type 1 and type 2 DM. The following text has been added to the discussion, as suggested. We also removed the reference 37 in the previous manuscript version.

“Our meta-analysis demonstrated only the benefits in delaying proteinuria among type 2 DM patients. This could be explained by a small sample size of type 1 DM patients (213 vs 97). Additionally, the pathophysiology of diabetic nephropathy in type 2 DM and type 1 DM patients is somewhat different. For type 2 DM, proteinuria could be caused by various etiologies including but not limited to insulin resistance, concomitant hypertension and obesity. One of the possible explanations would be that among type 2 diabetes there are pro-inflammatory cytokines generated from abundant adipose tissue as a part of obesity in type 2 diabetes. This inflammatory response leads to proteinuria among diabetic nephropathy. Omega-3 fatty acids help reduce insulin resistance as well as pro-inflammatory responses from adipose tissue. This effect might result in lower proteinuria compared to patients with type 1 diabetes which proteinuria is mainly through polyol, hexosamine, advanced glycation end product and protein kinase C (PKC) pathways (reference 35, 36). Nevertheless, any meta-analyses could not derive explanations for any mechanistic pathways or derive a hypothesis. Hence, future studies designed to examine mechanisms of omega-3 fatty acids on proteinuria or kidney functions are needed as well as to assess the effect of omega-3 fatty acids on inflammatory cytokines among type 1 and type 2 diabetes.”

For the sentence after that, we would like to raise the point that any meta-analysis could not derive or proof any mechanistic pathway.

We greatly appreciated the editors’ and reviewers’ time and comments to improve our manuscript.
